# Postings and transfers in the Ghanaian health system: a study of health workforce governance

**DOI:** 10.1186/s12939-017-0583-1

**Published:** 2017-09-15

**Authors:** Aku Kwamie, Miriam Asiamah, Marta Schaaf, Irene Akua Agyepong

**Affiliations:** 1 Ghana Health Service, Research and Development Division, Ministries, P.O. Box MB190, Accra, Ghana; 2 Achimota, Accra, Ghana; 3 Averting Maternal Death & Disability Program (AMDD) Heilbrunn Department of Population and Family Health Mailman School of Public Health, Columbia University, New York, USA

**Keywords:** Postings and transfers, Health workforce, Governance, District health systems, Ghana

## Abstract

**Background:**

Decision-making on postings and transfers – that is, the geographic deployment of the health workforce – is a key element of health workforce governance. When poorly managed, postings and transfers result in maldistribution, absenteeism, and low morale. At stake is managing the balance between organisational (i.e., health system) and individual (i.e., staff preference) needs. The negotiation of this potential convergence or divergence of interests provides a window on practices of postings and transfers, and on the micro-practices of governance in health systems more generally. This article explores the policies and processes, and the interplay between formal and informal rules and norms which underpin postings and transfers practice in two rural districts in the Greater Accra Region of Ghana.

**Methods:**

Semi-structured interviews were conducted with eight district managers and 87 frontline staff from the district health administration, district hospital, polyclinic, health centres and community outreach compounds across two districts. Interviews sought to understand how the postings and transfers process works in practice, factors in frontline staff and district manager decision-making, personal experiences in being posted, and study leave as a common strategy for obtaining transfers.

**Results:**

Differential negotiation-spaces at regional and district level exist and inform postings and transfers in practice. This is in contrast to the formal cascaded rules set to govern decision-making authority for postings and transfers. Many frontline staff lack policy clarity of postings and transfers processes and thus ‘test’ the system through informal staff lobbying, compounding staff perception of the postings and transfers process as being unfair. District managers are also challenged with limited decision-space embedded in broader policy contexts of systemic hierarchy and resource dependence. This underscores the negotiation process as ongoing, rather than static.

**Conclusions:**

These findings point to tensions between individual and organisational goals. This article contributes to a burgeoning literature on postings and transfers as a distinct dynamic which bridges the interactions between health systems governance and health workforce development. Importantly, this article helps to expand the notion of health systems governance beyond ‘good’ governance towards understanding governance as a process of negotiation.

## Background

A health workforce which is appropriately deployed and well-motivated is crucial to supporting health service delivery [1]. Postings and transfers – that is the decision-making and negotiation regarding where health providers work, and their geographic mobility within the health system – is an under-researched aspect of health workforce governance [2]. How and why the health workforce is deployed across geographic settings is foundational to the delivery of public services because it affects attainment of health goals like universal health coverage through issues of maldistribution, absenteeism, poor morale, decreased efficiencies, and lowered health system accountability. The limited evidence to date shows that postings and transfers are mediated by a variety of complex factors, such as public administration standards, labour market forces, political dynamics, professional power, human resource management systems and accountability mechanisms [3]. There is much literature from Ghana and other low- and middle-income countries identifying factors which influence staff preferences to take up or stay in posts, such as remuneration, career progression opportunities, facility infrastructure and social amenities (e.g., schools for children, employment for spouses, road networks, and accommodation) [4–11]. However, much of this work has focused on broader issues of attraction, motivation and retention, with very minimal attention paid to understanding the actual dynamics of the posting and transfer process itself. What is missing in our understanding of postings and transfers is the complexity of negotiations between the one posting or transferring and the one being posted or transferred. These negotiations themselves are complex because they magnify issues of governance: they reflect potential flashpoints between individual and organisational goals which are embedded in broader policy contexts, health system organisation, and power dynamics.

The content of the concept of health systems governance has evolved over time from regulation and stewardship to greater acknowledgment of the importance of leadership [12, 13]. While the concept of governance in health continues to emerge, the literature remains lean and primarily normative, that is, there is little empirical evidence from real-life systems on ‘what is’ governance, rather than idealised statements of ‘what should governance be’. Recent systematic review evidence notes that ‘good’ governance mechanisms linked to improved health outcomes include (1) responsive health system decentralisation, (2) transparent and participatory health policy-making, (3) increased community engagement and (4) increased social capital [14]. Barbazza and Tello [15] note that despite the lack of consensus surrounding the concept of governance in health, there appears to be convergence around governance in health as a “set of processes (customs, policies or laws) that are formally or informally applied to distribute responsibility or accountability among actors in a given system”. This links closely with Bossert and Brinkerhoff’s definition of governance as “the rules that distribute roles and responsibilities among government, providers and beneficiaries and that shape the interactions among them. Governance encompasses authority, power and decision-making” [16]. Both of these definitions acknowledge the co-existence of formal and informal rules and norms which guide behaviour.

To date, the literature has done little to draw out the interactions between overall health system governance and health workforce development [17]. Health sector reforms and health workforce policies have often been developed separately, the result being limited evidence and understanding of how these shape health workforce governance [18–25]. The objective of this article is to explore the formal and informal policies and processes which underpin posting and transfer practices at district-level in two rural districts of the Greater Accra Region of Ghana. This has relevance for the design and implementation of appropriate posting and transfer policies sensitive to contextual conditions. More broadly, it opens a window into the everyday practice of governance as a process of negotiation within health systems.

## Methods

### Semi-structured interviews

The first two authors collected data in two rural districts of the Greater Accra Region of Ghana (District A and District B) from August 2015 to February 2016. These districts were identified based on the researchers’ previous work in the region, and on the basis of existing trust relationships. Due to their rural nature, the districts were further purposively selected for this study to build understanding on the particular challenges of postings and transfers in rural settings. Given the perceived potentially-sensitive nature of the findings, we anonymised the districts. Audio-recordings were not made of the interviews for the same reason. Interviews were conducted by the research pair together; one conducted the interview and the other took detailed notes.

The development of semi-structured interview guides was informed through a non-exhaustive review of the human resources for health literature. Interviews sought to understand: (1) how the postings and transfers process works in practice; (2) factors in staff and district manager decision-making about postings and transfers; (3) personal experiences in being posted; and (4) practices regarding study leave. We investigated study leave specifically because it emerged as a potentially important dynamic from observations in prior formative research (unpublished data).

Semi-structured interviews were conducted by the research pair with managers at the district health administration (DHA), and frontline staff across all facility types in both districts: district hospital or polyclinic, health centres and Community-based Health Planning and Services (CHPS) compounds which offer basic health services at household level. In both districts, the key managers directly involved in the postings and transfers process were identified as being the district director of health services, deputy director of nursing services, and administrator. In each district four interviews were conducted (the above-listed managers, plus one additional manager not directly responsible for postings and transfers), a total of eight district managers, reflecting two-thirds of the management team in each district.

Eighty-seven frontline staff were interviewed (43 from District A and 44 from District B). In District A, 21 of 62 polyclinic staff were interviewed based on availability: this included the full complement of those on duty during the days of interview (not counting those on leave or off-duty, with the exception of labour ward staff who were not included so as not to interfere with service delivery). Fourteen of 25 health centre staff were interviewed (the total complement on duty on the days of interview). Two of the district’s six CHPS compounds were randomly selected (one near to district capital where the polyclinic is located, and one remotely located from the district capital). Four staff were interviewed at each CHPS compound, reflecting the total staff complement of each. In District B, 12 of 36 hospital staff available at the time of interview (the hospital runs three shifts) were interviewed. CHPS compound staff were opportunistically interviewed at a CHPS training workshop: 31 of 36 CHPS staff were interviewed from all nine CHPS compounds. Interviews were held at the larger health facilities over a 2-day period each. The bulk of staff interviewed were community health nurses, enrolled or general staff nurses, and midwives (see the demographic data for both districts in Tables 1 and 2 below). We aimed for a representative sample in both districts.

**Table 1:** Demographic data for district managers

District managers	District A	District B	Total	% of all respondents
	(*n* = 4)	(*n* = 4)	(*n* = 8)	(100%)
Mean age in years	42.5 (SD = 9.11)	40.7 (SD = 9.25)	-	-
Mean years in post	2.33 (SD = 0.29)	4.45 (SD = 1.89)	-	-
Mean years in GHS	16.92 (SD = 8.80)	17.7 (SD = 9.49)	-	-
Sex				
Male	1	1	2	25%
Female	3	3	6	75%
Professional classification				
Medical doctor	-	1	1	12.5%
Physician Assistant	-	-	-	-
Midwife	-	-	-	-
Public Health Nurse	1	1	2	25%
Disease control officer	-	1	-	-
Nutrition officer	1	-	1	12.5%
Health information officer	-	-	-	-
Pharmacist	1	-	1	12.5%
Administrator	1	1	2	25%
Accountant	-	-	-	-
Other	-	-	-	-

**Table 2:** Demographic data for frontline staff

Frontline staff	District A	District B	Total	% of all respondents
	(*n* = 44)	(*n* = 43)	(*n* = 87)	(100%)
Mean age in years	33.93 (SD = 11.58)	30.81 (SD = 8.18)	-	-
Mean years in post	2.94 (SD = 2.72)	2.73 (SD = 4.98)	-	-
Mean years in GHS	8.95 (SD = 11.27)	5.68 (SD = 6.28)	-	-
Sex				
Male	5	4	9	10%
Female	39	39	78	90%
Professional classification				
Medical doctor	1	-	1	1%
Principal nursing officer	1	-	1	1%
Midwife	5	7	12	14%
Public Health Nurse	2	-	2	2%
Disease control officer	-	1	1	1%
Nutrition officer	1	-	1	1%
Superintendent enrolled nurse	1	-	1	1%
Community health nurse	14	19	33	38%
Clinical nurse	0	2	2	2%
Enrolled nurse	10	10	20	23%
General staff nurse	9	2	11	13%
Registered general nurse	-	2	2	2%

Interview notes were handwritten and converted into digital transcripts on the same day. Interview data labelled by unique identifier were cleaned and coded manually in an inductive manner. To ensure rigour in the analytic process, we triangulated the data across sources (document review and interview data) respondent types (managers and staff), and districts (A and B). To reduce researcher bias, daily reflections during data collection between the first two authors took place. Interpretations of findings were validated by feeding them back to interviewees to check for their accuracy.

## Results

### Context for postings and transfers in Ghana

The Ghana Health Service (GHS) is the public sector service delivery agency of the Ministry of Health (MOH). The GHS was established by the Ghana Health Service and Teaching Hospitals Act (525) of 1996, which created an agency model for the MOH. The GHS is administratively decentralised down national, regional, district and sub-district lines. In terms of human resources management, the MOH has a Human Resources Directorate with overall responsibility for the nation’s health workforce planning, administration and development across its different agencies (which apart from the GHS includes regulatory, purchaser and training agencies). Within the GHS itself, a Human Resources Directorate at national-level headquarters is responsible for training, planning and management functions of the public sector health service delivery workforce it controls.

The majority of health staff are employed in the public health sector; the most recent figures (2009) indicate 46,040 staff on the public payroll [26]. Professional nurses make up the greatest proportion of health workforce in Ghana (i.e., 26.8%); this is followed by enrolled nurses (25.4%), community health nurses (18.4%), and midwives (12.8%) [26]. Though the Greater Accra Region is the second most populous region in the country, being the capital city region, it attracts the greatest numbers of staff, and has the highest proportion of professional nurses, midwives and community nurses in the country.

In terms of the districts under investigation, District A has a population projected at 73,000, (2014) made up of rural and peri-urban areas. Residents mainly engage in subsistence and some small-medium scale commercial fishing and farming. Of note is the existence of a large private university in the district, which attracts many students (District A Annual Report, 2014). District B is the district with the largest land mass in the region, and has a population projected at 60,000 made up of 167 scattered communities. The district is considered to be disadvantaged, with most inhabitants engaging in subsistence farming and fishing (District B, Annual Report 2014).

### Demographic data

Demographic data for district manager and frontline staff respondents are summarised in Tables 1 and 2 below:

We found that interviewed staff were young (mean age 34; mode = 27), in District A and mean age 31 (mode = 28) in District B, with the majority of staff in their first posting, having served less than 3 years on average across both districts. Ninety percent of staff respondents were women, reflecting the fact that most of them were nurses, and nursing is a female-dominated profession in Ghana. Responses in District B largely mirrored those from District A, with the exception of a particular dynamic around study leave, described in more detail below.

### Postings and transfers: formal policies

Formal rules for decision-making authority for postings and transfers are cascaded down MOH and GHS lines [26]. Each level, from MOH headquarters to GHS headquarters, to regional, district and facility levels has a different scope of decision-making authority regarding the posting and transfer of employees, generally related to the mandate of that level. Thus, MOH as the coordinating body for the entire health sector determines agency staff allocations, and post staff to its agencies (such as the GHS). MOH has no formal posting and transfer powers within the agencies themselves. GHS headquarters determines staff quotas at regional level and posts to regions, but has no posting and transfer powers to specific districts. GHS-HQ must, however, be informed of the final postings and transfers within-region as part of its monitoring of staff distribution. If staff want to move from one region to another, they have to seek release from their region of work to GHS-HQ, and GHS-HQ confirms that there is a vacancy in the region the staff desires to move into prior to posting them. A region can refuse to release or accept staff. Similarly, the region posts to districts and is not supposed to interfere with facility posting within-district. Again, the district can refuse to release or accept staff.

The draft policy on postings^1^ (2015) identifies a policy goal of equitable staff distribution, with a focus on posting staff to where their services are needed, according to district plans. The policy distinguishes between postings at headquarters, postings across and within regions, and across and within districts. Inter-regional postings are identified on regional needs and are the responsibility of either the director-general, director of human resources, or regional directors of health services, depending on the category of staff. Postings across districts are the responsibility of regional directors. Postings within districts are the responsibility of district directors. Therefore, it appears that the bulk of posting and transfer powers exists somewhere between the region and district. Staff distribution is meant to be done on the basis of need, geographical access and equity, and the principle underlying posting is that staff are to be distributed solely on a basis of vacancies, and they are to “be done with fairness and transparency”. The policy further recognises that the lack of differential incentivisation across rural and urban settings contributes to maldistribution of staff. The policy operates on a principle of ‘train and retain’, meaning that new graduates from MOH training institutions are required to serve in the regions where they were trained. Procedurally, it is the regional director who issues posting letters to staff with copies to the receiving district where staff are meant to report to the district director. The district director then posts staff to sub-district facilities. Heads of facilities report the assumption of duty to the district when staff have reported there. The policy denotes consideration of where spouses work as a privilege, not a right.

### Postings and transfers: real practices

It is generally understood that while the rules are generally adhered to, there are informal lobbying mechanisms by which people get around the rules based on ‘who you know’ at which level, and also that the strongly hierarchical nature of the health system makes authority at lower levels hesitant to confront authorities at higher levels. From the data three themes emerged: 1) differential negotiation-spaces at regional and district level surrounding postings and transfers; 2) lack of clarity of staff on conditions of service for transfer or study leave eligibility; and 3) a sense of unfairness in the posting and transfer system from the perspective of district managers and staff alike.

In practice, staff reach the district in a variety of ways. There are two negotiation-spaces during the posting process. The first occurs at the region-level interview. First, staff are given a GHS posting form to fill and submit. They are then called for interview. Staff largely reported that they are posted to the region where they were trained. However, there were several cases where the regions were “full”, i.e., had reached their quotas for staff of particular cadres. In those cases, staff were able to choose another region, or were assigned a region directly. About half of staff reported that during the regional interview they were given the option of choosing three districts of their preference. If a staff is married they can provide their marriage certificate to support their request. However, choices are not met in every case. Others reported being assigned a district directly, without option. From our data it appears that a combination of vacancies and whether or not a particular staff cadre is in need influences regional managers in making posting decisions, however this does not appear completely systematic, i.e. there is no evident pattern by which staff may be given district options or are assigned. Regional managers use their discretion. Of those staff who were assigned there was lack of understanding and even negative feelings towards the nature of their assignment:

*“I was assigned. We were in a long queue, and when it was your turn you will be told where you are being posted to. At the time I had braids in wine colour [note: deemed unprofessional], so when the woman saw me she kept saying that I will be posted to [District A], as if it is a punishment.” (Polyclinic staff nurse, FS-036)*

Of those interviewed staff who were given options, the single-most important factor in their choice of district was accommodation availability, particularly in rural areas where staff are less likely to have relations or friends. Other factors which influenced their choice of district were varied and ranged from marital issues (desiring to be near spouses), parental/familial issues (desiring to stay near ailing parents or extended family, or desiring to move away from family to gain independence – this was especially important for younger staff in their first post), and greater exposure to work tasks in a rural setting. Staff also heavily relied on their informal networks (colleagues and extended family) to advise on the particularities of a district to inform their choice. For example, a district might be desirable because of low rents, or perceptions of low workload at the facility.

The majority of interviewed staff accepted their postings without dispute because they were primed in school that they may be posted anywhere. Others also felt a sense of duty to serve. Still others believed that it simply was not allowed to refuse a posting, or that refusal will lead to unemployment, partly because they have observed this happen to other colleagues. Our findings reflect the fact that the majority of interviewed staff did not feel empowered to refuse the district to which they were assigned. When staff did feel emboldened to refuse postings, a key reasons was when they had observed other colleagues having had the ability to change their postings. These changes were possible regardless of whether the health worker had someone to lobby on their behalf or not:

*“If you go back to Region for reposting it would have been done. I know people who had theirs changed yet they didn’t know anyone at Region.” (Health centre enrolled nurse, FS-015)*

The second negotiation-space occurs when staff report to the DHA. Districts receive new staff via two means. The first is that the DHA scans the needs of the district and then lobbies the region for them to assign staff, and are successful. The second is when the region sends staff that they have received to the district, often straight from the training institutions:

*“You have two scenarios, one where you know you have deficiencies and you lobby. You let them [Region] know you have deficiencies and you continue to follow up. Or the other is when you have someone sent to you. The second one is based on school-finishing. It is more predictable, but the scenarios intertwine because when they finish school they come, so if you lobby, then Region keeps you in mind.” (District Manager 005)*

Staff are to appear at the DHA they have chosen or have been assigned to with their posting letters. Once at the DHA, staff are interviewed by the district director and deputy director of nursing services. Staff are then assigned to sub-districts; certain cadres, such as community health nurses which are numerous, ballot for placement, which involves placing their names on pieces of paper for random selection for the sub-district facility they will be posted to. Interviewed staff noted that there is little room for negotiation at the district:

*“No, there is no amount of words you will say or cry that they will change it.” (CHPS Community health nurse, FS-085)*

District managers largely concurred that in their posting decisions the needs of the district took priority over posted staff’s personal factors. While the DHA did try to accommodate staff by balancing factors of staff qualifications, marital status, language and housing accommodation, district managers indicated that these do not override the needs of the district:

*“Staff choice does not usually prevail, especially new staff. Sometimes you have to sit them down and talk to them. You negotiate with them and give them a timeframe to go and try the place, and if they are still unhappy about it after then they can change. But most at times they don’t want to.” (District Manager 002)*

Where there appears to be even less negotiation-space is around transfers (all-types within and across districts and regions). There exists a perception that transfer was most often employed more punitively than as reward. Both district managers and staff shared this view. Transfers often came unexpectedly, were disruptive, and staff would simply be informed by the DHA that they were being transferred with minimal consultation. Many staff who had been transferred complained that they were given little time to move (in one case, just 1 week), and in a context of limited accommodations, especially when rents are paid 1–2 years advance without refund, this was particularly challenging:

*“Transfer – you will be there and will be notified that you are being transferred. But you have rented a room and then your money will be lost to the landlord. The DHA don’t care. They tell you: you have to go.” (CHPS enrolled nurse, FS-076)*

*“If [staff] output is not good, if it is non-performance, it results in a transfer” (District Manager 006)*

In practice, staff are eligible to apply for transfer after 5 years. If they are able to find someone to swap with them then they can go sooner, however the administrative procedures mean this can take several months. Half of the interviewed staff indicated they were not satisfied in their current posts.

### Explaining gaps between policy and practice (1): ambiguous conditions of service

The context in which postings and transfers occur is one dominated by a lack of staff awareness of conditions of service. As such, staff relied on their social networks for information, which results in much hearsay. Many interviewed staff indicated that they were unaware of any postings and transfers policy. Interviewed staff also varied in their belief that eligibility for transfer occurred after 2, 3, 4, 5, or 6 years. One major issue was the lack of policy consistency across urban and rural settings. For instance, it was rumoured amongst staff that serving terms were shorter in other regions of the country as compared to the Greater Accra Region. Furthermore, though District A and B are rural districts, many staff complained that because they are located in the capital city region the rural designation (which is supposed to confer an accelerated transfer process) is unevenly applied.

### Expanded example: study leave

One of the main issues for staff postings and transfers is related to study leave. Nearly all interviewed staff indicated that they have, or intend to apply for study leave. Only a handful indicated their intention to return to the district they are currently serving post-qualification. In 2013, the years of service eligibility to be able to apply for study leave was increased from 3 to 5 years. A memo dated May 2015 from GHS headquarters through the Regional Health Directorate directed study leave quotas for various training programmes across all regions (meaning that limitations had been placed on particular courses of study for eligible staff, thus increasing the competitiveness of getting study leave approval). Many interviewed staff reported disappointment with the policy change. More critically, staff had signed a bond during school to serve in their posts for 3 or 4 years before being eligible to apply for either transfer or study leave, and there is widespread lack of clarity amongst interviewed staff as to whether the bond they signed or the new policy holds. The new service term of 5 years was viewed universally by interviewed staff as too long:

*“In school we signed a bond of 3 years. No one has explained what happened to the bond. The majority of CHNs are women. Those that have a vision – if they consider marriage, upgrading themselves, childbearing – it prolongs everything you want to do.” (Polyclinic Community Health Nurse, FS-040)*

District managers noted the difficulties that the new policy had wrought:

*“Because more people are going [to school] now, so they bumped it from 3 years. People don’t understand, staff want to go to Region because they didn’t believe [that the policy had changed]. Region also asks us whether we are explaining it well. It is a sudden change. I think 5 years is too much. It was a blow to some, because they are thinking that this is what happened to my seniors, so when it is my turn now [I can also go]. So now it is if you know someone: “my girl is here, please sign her letter…”. Even if you refuse to sign, she can still get someone at Region to sign it so that they are released.” (District Manager 004)*

District managers also explained that though 5 years of service are required prior to study leave eligibility, years of service also depended on the course being applied for, and if there is a priority need for certain cadres, staff applying for those cadres can be ‘fast-tracked’.

Interviewed staff also complained that study leave approval could be delayed in cases where senior staff were entitled to have their leave approved first, so even if years of service have been met, study leave could be rejected:

*“It depends if someone higher than you – meaning if they have been here longer and if they are a higher grade than you – then they have to go first, so you are delayed.” (Health centre staff nurse, FS-011)*

Study leave with pay is dependent on staff attending an approved list of training institutions, as well as an approved list of training programmes. The procedure for study leave is that staff submit a letter of intent 1 year in advance of when they would like to go on study leave. However, interviewed staff reported that it was not clear whether they are to submit in year 4 with intention of attending school in year 5, or submit in year 5 with intention of attending school in year 6. Interviewed staff also reported feeling limited by rigid career paths. In practice this meant that staff wanting to shift career paths or study other programmes may not have their leave approved, or may take leave without pay, and risk their qualifications not being recognised as part of their promotion. Some staff use their personal leave to take courses:

*“I wanted to further my education. I went on leave without pay. I already had admission so I left before my approval letter was ready. GHS doesn’t recognise the course, so promotion will be a problem for me. I heard about it before leaving so I prepared my mind. I returned to my same post.” (District hospital Community Health Nurse, FS-046)*

Some staff attend school clandestinely, especially in District A where a private university is located. District managers confirmed that it was the case that some staff wanted to be posted into the district specifically so that they could attend the university. District managers also mentioned the long approval processes which further complicate study leave:

*“One of the challenges is the processing time for study leave. You find that staff will leave to the region for study leave, by the time it takes to go up the levels of approval, the back and forth, and some time is needed from HQ – you realise that staff are already in school before approvals come. Sometimes it takes even one year.” (District Manager 006)*

Interviewed staff also compared themselves to their Ghana Education Service (GES) colleagues posted to the same district, and were dissatisfied that GES staff were allowed to attend distance or sandwich courses with pay (which is not allowed by the GHS), thus enabling GES staff to access promotions more quickly:

*“What I know is that in school we sign a bond but it is for 4 years. I learned when I arrived that it is 5 years. If it changed it should not affect us but those coming [in]. When you compare it with the way of schooling with Ghana Education Service, it is not fair because we are not allowed to study while working, but the Ghana Education Service does. So you are waiting for 4 years working, doing nothing unless they give you study leave. You start with [the] teachers, you come to the district you all have diplomas, and before you hear they have degrees, masters…” (Health centre Community Health Nurse, FS-021)*

### Explaining gaps between policy and practice (2): perceptions of system unfairness

The lack of policy communication, and thus staff clarity surrounding conditions of service results in a sense of unfairness in the system from staff and district managers alike. However, because of the greater negotiation-space (in the case of postings) at regional-level as compared to district-level, interviewed staff appear to trust the region more, viewing them as more favourable to their preferences:

*“I will be eligible in 2018 according to them. We signed a bond of 4 years. They are now saying 5 years. I am bound by what I have signed, but I don’t know what the DHA will honour. The difference is if you go to region they will listen, but DHA will think of what they need.” (Health centre general nurse, FS-067)*

District managers and staff alike complained that this can sometimes interfere with staff negotiations:

*“It’s only when they go behind your back if they know someone – like at national-level or an MP. They do lobbying up there already before they even come. When they start crying or are rude, if you are not fast they will go to Region and tell a different story to get a new letter. So now I call directly the Regional Health Directorate and Chief Nursing Officer and they give their assurance that they will turn them back.” (District Manager 005)*

*“Those who refuse, sometimes it’s the fault of the Region. Region will instead call the district and interfere by instructing them what to do with a certain person. They shouldn’t instruct, they should just post.” (CHPS Midwife, FS-023)*

## Discussion

The objective of this study was to explore the formal and informal policies and processes which underpin posting and transfer practices at district-level in two rural districts of the Greater Accra Region of Ghana. We sought to investigate the rules which govern actions of district managers and frontline staff in postings and transfer practice. This was justified from a policy and practice perspective to potentially better inform the geographic deployment and mobility of health workers to address issues of equitable distribution, and from a research perspective to buttress an under-researched and distinct aspect of the human resources for health literature.

Overall, it appears that while the cascaded authority for posting and transfer decision-making (from MOH to GHS-HQ, regional and district levels) is well-laid out in theory, in reality it is the differential negotiation-spaces at regional and district level which guide postings and transfers practice – namely through informal lobbying, and managerial discretion (governed by managerial decision-space). The differences exercised at regional and district level have different effects on staff perception of how they are handled by the system. Decision-space – that is the range of choice available to local actors – is a concept which has been well-explored in the literature [27–29]. Decision-space is influenced by 1) the resources managers have access to, 2) their managerial capacities, 3) the accountability mechanisms regulating them, and 4) their operational context [30]. Our previous findings on district-level decision-space in Ghana have shown the overall operational context of the health system as being one of strong hierarchical authority and resource uncertainty at district level, meaning that district-level management is less responsive at local levels because it is focused upwards on meeting national-level demands and resource dependency, rather than downwards on health facilities and communities [31]. Our findings here demonstrate how bureaucratic functions get translated through policy implementation and how people respond; that negotiation is not a static occurrence, but rather an ongoing one. From our findings, this can be seen in the fact that there are multiple spaces for negotiation, occurring in this case at both the regional and district levels, and that these spaces do not have clear ‘rules’ which guide them, meaning that they can be ‘tested’, that is people can try them to achieve a given result (which may be positive or negative). These negotiations are embedded within broader policy contexts of systemic hierarchy and implicit resource dependence on higher levels [32]. It is not surprising that in a context of limited awareness of the formal written postings and transfers policy, lacking clarity on policy goals results in hearsay and misunderstandings as well as perceptions of an unfair system. It is clear that when policy clarity is lacking, staff try to make sense through observation and information gathered from their informal networks.

There are clear tensions between organisational and individual goals. In this case, it appears that the organisational goals ‘win’, because managers are able to prevail in their decisions to post and transfer staff (as compared to staff prevailing in their choices of post based on accommodation, familial or experiential preference). We also noted differential levels of trust between staff and regional- and district-level managers, as health workers perceive greater negotiation spaces at regional-level as compared to district-level. The increase in eligibility years for study leave, coupled with insufficient policy communications surrounding the policy change, and the issues of suspended bonds has also had major implications for the levels of trust staff have in the system itself.

The literature on postings and transfers remains nascent, and our findings contribute to this literature. There are lessons from other countries with which our findings converge. For instance, in Pakistan, Collins and colleagues note the high circulation of staff in the health system related to poor implementation of formal rules and the nature of public sector control [33]. In Nepal, career paths were seen to be important to keeping staff in service [34]. Research in India found that the tensions between organisational and individual goals can be difficult to align in postings and transfers, particularly in contexts of ambiguous or ‘pliable’ policies and staff shortage, which makes manipulation of the system from the perspective of either managers or staff possible [35]. In Nigeria, Abimbola and colleagues point to three functions of postings and transfers, including its routineness improving system performance, or punishment of staff, and how the lack of clear policy leads to differential postings and transfers practice which does not always lead to equitable distribution of staff [36]. From our findings, given the lack of policy clarity from the perspective of staff, and the multiple and differential negotiations surrounding postings and transfers, it is not apparent that organisationally, the GHS is meeting its policy goal of “distributing staff *solely* on the basis of vacancies […] done with fairness and transparency”. It is of note that district managers view themselves as receivers of staff from regional-level, and identify the region as interfering in district-level decision-making at times, whereas frontline staff view the regional-level as more amenable to their personal situations. When being posted, staff have some room to negotiate; however, in the case of transfers they have little room for negotiation. Staff feel disempowered by their inability to challenge decisions. District managers appear to be committed to the stated objectives of the postings and transfers policy, and may possibly be acting to control the decision-space they do have, in a context where they feel they have too limited control over resources. This underscores the delicate tension which exists between the interests of the person and the interests of the system.

Our study contributes to the expanding notion of health systems governance. This study usefully illustrates the influence of people in the ongoing negotiations which govern decisions on how the health workforce is deployed; this goes beyond the structures of ‘good’ governance, or the set of applied processes meant to guide decision-making. Secondly, our study helps to fill the gap in the literature bridging overall health system governance and health workforce development. By examining postings and transfers as a dynamic process, we distinguish it from the existing, static evidence on staff attraction and retention factors. Postings and transfers are complex, and shaped by the complexities of what governs negotiations, which of course includes staff attraction and retention and more.

The young age of staff in this study (the bulk of whom are in their first posting) does not reflect the broader composition of GHS staffing. This suggests high turnover in these rural districts, that new staff are entering and do not stay in the district. The majority of staff do not refuse postings, yet staff appear to be using study leave as a legitimate exit strategy. This makes sense in an organisational culture of strong hierarchy, and a cultural context of compliance.

From a governance perspective the distribution of roles and responsibilities for health workforce decision-making authority and power influences the interactions between regional and district management and staff performance [16, 37]. A greater degree of alignment between regional and district levels would support health workforce functioning overall. It is also clear that greater attention must be paid to the ‘software’ (i.e. the ideas and interests, norms and values, power and relations) [38] which oil the ongoing negotiations of postings and transfers.

We note some limitations in our study. First, the study does not include regional or national level perspectives. These would aid understanding about the ongoing negotiation of posting and transfer across the health system. Secondly, considerations of gender in low- and middle-income country health workforce policies have also been lacking [39]. Most attention to gender in health has reflected service demand and access aspects, with less attention paid to gender in relation to staff service conditions, motivations and hidden biases in career progression [40]. Though the study does not explicitly investigate gendered issues of postings and transfers, we do note that 90% of the respondents were women, and their responses address marital and childbearing issues (for example in the case of study leave). Gender is a particularly important contextual factor in health workforce governance, not least because a majority of the health workforce are women and are concentrated at the lower levels of the health system [41]. Ghana’s policy on gender [42] admits its own failings and lack of structures and mechanisms to address gender concerns within the health workforce. This presents a potential area of future research.

## Conclusions

In a context of ambiguous conditions of service, frontline staff rely on information gathered through informal networks to make sense of the postings and transfers system. Staff do not necessarily refuse rural postings, but seek legitimate exit routes through study leave. Insufficient communication surrounding changes in postings and transfers policy, and the limited negotiation-space at district-level as compared to regional-level exacerbates staff perceptions of an unfair system. There is great need for policy consistency and clarified information to better align staff and system goals.

## Abbreviations

CHPS: Community-based Health Planning and Services
DHA: District Health Administration
GES: Ghana Education Service
GHS: Ghana Health Service
MOH: Ministry of Health

## Footnotes

1 While the Ghana Health Service Policy on Postings (2015) is titled ‘postings’ only, it in fact includes sections on inter-regional postings, intra-regional postings, which are ‘transfers’ in this case. The policy refers to transfers as movement out of the Service to another Public Service Organisation.
